# Effectiveness of a text-messaging intervention on intuitive eating: a randomised controlled trial

**DOI:** 10.1017/S1368980023000939

**Published:** 2023-08

**Authors:** Batoul Manana, Claire El-Jor, Joelle Abi Kharma, Nadine Zeeni

**Affiliations:** Department of Natural Sciences, School of Arts and Sciences, Lebanese American University, 36-Byblos, Lebanon

**Keywords:** Intuitive eating, Self-compassion, Perceived stress, Eating behaviour, Randomised controlled trial

## Abstract

Intuitive eating (IE) is a concept based on mind–body integration of instinct, emotion and rational thought, to improve eating behaviours, physical and mental health. Several attempts were developed to teach IE principles including text-messaging interventions given that they are easy to implement and low cost.

**Objective:**

The present study aimed to assess the effectiveness of a 5-week text message-based IE intervention on IE, while correcting for perceived stress (PS) and self-compassion (SC).

**Design:**

A randomised controlled trial.

**Setting:**

Online, in Lebanon.

**Participants:**

Adults (*n* 195) were randomised into one of three groups: the active IE group receiving IE-related messages with a practice exercise, the passive IE group receiving only IE-related messages and the control group receiving general health-related tips. Ten messages were delivered over 5 weeks. Two follow-ups were made: directly post-intervention and 7 weeks later. Baseline data and follow-ups included demographics, nutrition-related variables and measures of IE, SC and PS.

**Results:**

Results indicated improvements in IE scores in the two intervention groups (*P* = 0·05), with the passive IE group showing the most improvement. Also, a significant improvement in SC (Λ = 0·88, F (2, 63) = 4·40, *P* = 0·01) and reduction in PS (Λ = 0·86, F (2, 63) = 5·21, *P* = 0·008) were observed in the active IE group.

**Conclusion:**

Text-messaging interventions might be efficient in enhancing IE. Results shed light on the need for further large-scale interventions that use visual aids and provide practical guidance to teach IE, while further characterising the relation between IE, SC and stress.

Healthy eating has evolved beyond dieting and caloric restrictions. While traditional weight loss methods have shown success over a short period, they were also associated with poor weight maintenance, health issues and deterioration in quality of life of individuals^(1)^. For that, intuitive eating (IE) was created as a novel concept that allows individuals to connect to their own hunger cues, where only themselves can determine when and what to eat, by listening to their body needs^(1)^. Accordingly, IE is an attempt to create an enjoyable relationship with one’s self on the one hand and with food and eating on the other hand. It is possible by focusing on gentle nutrition, an anti-diet approach in which nutrition affects health, and joyful movement, showing respect to one’s body and being kind to one’s self while choosing to move your body without rules in any exercise or non-exercise form^(1)^.

IE is an adaptive form of eating that guides eating behaviours according to internal hunger and satiety cues, rather than external emotional drives or dieting guidelines^(1,2)^. It is defined as ‘the dynamic process-integrating attunement of mind, body and food’ and is based on ten principles^(1)^. This approach aims at enhancing the mind–body connection and reinforcing body gratitude, through the adoption of three core beliefs. The beliefs are reliance on internal rather than external hunger cues to determine what, when and how much to eat, the unconditional permission to eat unlimited amounts of food and listening to hunger cues without ignoring them for any reason^(2,3)^.

Several internal and external factors that can either restrict, enhance or inversely correlate with the ability to eat intuitively have been identified, among which perceived stress (PS), self-compassion (SC) and BMI^(4–6)^. More specifically, recent literature showed that IE practices were associated with lower PS levels, dieting and disordered eating, along with enhanced SC and emotional management as well as reduced BMI, with the latter being correlated with IE via mediators like the intake of specific food, binge eating frequency and speed of eating^(6–13)^. However, SC and PS tend to influence each other inversely. Both intervention studies and observational analyses documented that increasing SC can decrease PS, which can also lead to an improvement in BMI^(14–17)^. This might indicate a positive effect of SC on IE, as described previously, via a reduction in PS.

In a recent systematic review of interventions investigating mindful eating and IE, neither diet quality nor energy intake was found to be significantly improved in the intervention groups, highlighting the need of further research of better study design qualities^(18)^. Aside from energy intake and diet quality, several studies assessed different IE interventions and their effect on weight, BMI, self-esteem and other health-related outcomes. The Health at Every Size programme incorporated IE principles through twenty-four guided weekly sessions and was compared with a standard diet programme^(19)^. Health at Every Size group participants showed better size acceptance, less dieting behaviours and more body signal awareness and response^(19)^. Another study assessed the ‘Mind, body Food’ web-based interventions, to teach IE to middle-aged women^(20)^. The intervention was delivered in twelve online modules that included IE discussions and guided-audio activities. After the intervention, enrolled women had improvement in IE, physiological flexibility and general mental health. Moreover, a recent study examined the effectiveness of an IE intervention through text messaging *v*. emailed handouts on IE, stress and self-efficacy of college students^(21)^. The texting programme was 5 weeks long and consisted of ten messages, based on the ten IE principles. On the other hand, the email programme was a single PDF handout that contained the messages’ content all at once. Results showed that the text-messaging intervention significantly improved IE in the text-messaging group, whereas the control group’s IE scores had not changed. Therefore, IE interventions delivered through text messages could be a promising and cost-effective approach to promote IE. However, the study did not assess participants’ engagement with the messages. Besides, previous work has shown that effective eating behaviour-targeted text-messaging interventions require active rather than passive involvement from the participants^(22)^. For example, childhood obesity was targeted by a messaging intervention that involved interactive questions and encouraging feedback messages to remind the participants about the learnt skills^(22)^. Hence, active engagement in the text messages could improve the learning of the content.

To the best of our knowledge, no studies have evaluated the short- and long-term effectiveness of an active and a passive text-messaging intervention on IE while considering IE barriers and enhancers among the general population. Hence, the present study aimed at assessing the effectiveness of a 6-week text message-based IE intervention on IE, while correcting for SC and PS level. Accordingly, we hypothesised the following:

H1: The 5-week text message-based IE intervention will be effective in enhancing IE in the short and longer term.

H2: The active text-messaging IE intervention will be more effective in enhancing IE compared with the passive intervention.

## Methods

### Study design

This randomised single-blinded controlled trial followed a parallel design, it had three arms and participants were recruited online via sharing the advertisement material and the link to the consent form on social media platforms (Facebook, Instagram and WhatsApp®). The study is registered in the Lebanese Clinical Trials registry (LBCTR2022045031). The questionnaires were filled via Google Forms, and the intervention content for all the groups was communicated via WhatsApp®. A detailed description of the study methodology is provided in the online supplementary material.

### Participants

Men and women, living in Lebanon, aged between 18 and 50 inclusive, who have a smart phone with an active WhatsApp® account were included in the study. Phone numbers of the participants were obtained upon online consent. Participants enrolled in weight loss programmes or taking medications that affect weight/appetite were excluded from the study. The sample size was calculated based on the effect size of the web-based IE intervention study^(20)^, which is similar to this study in terms of outcome measure using the same scale, and its online nature. The mean IES-2 change was 0·94 ± 0·67 indicating a moderate-high effect size; accordingly, sixty-five participants were needed per arm. Since there were three arms, a sample size of 195 participants was needed. Randomisation took place between February 2021 and November 2021. Upon consent, participants were randomised to an active intervention group who received IE-related messages with practice exercises adapted from the IE workbook^(23)^, passive intervention group who received only IE related messages and a control group who received general health-related messages. Randomisation was based on permuted block sampling with six participants per block with an allocation ratio of 1:1:1. The participants were blinded for allocation and study objectives until the end of the study period.

### Procedures

Recruitment and data collection were initiated online using the snowballing technique. Upon consent, participants were able to fill in the baseline survey (T0). Then, each consented participant received ten messages over 5 weeks, at a rate of two messages per week, that is, every Monday and Thursday starting the first week after consent. An additional online survey (follow-up 1, T1) was administered right at the end of the intervention (i.e. after week five) and then again 5 weeks post-intervention (follow-up 2, T2). Then, after follow-up 2, the participant irrespective of the allocated group received a debriefing video that explains IE principles with practical tips and examples. The surveys, messages and videos were provided in both Arabic and English.

### Measures

The primary outcome of the study is IE. It was assessed through the Intuitive Eating Scale 2 (IES-2) embedded in the surveys. It is a 23-item, 5-point Likert self-reported scale used to assess the eating habits of individuals over the past month. It is composed of four subscales: the unconditional permission to eat, eating for physical rather than emotional reasons, dependence on hunger and satiety cues and body-food congruence^(3)^. The secondary outcomes are PS and SC, and their scales were also embedded in the survey. SC was assessed using self-compassion scale (SCS), which is of 26 items, 5-point Likert scale^(24)^. PS was measured by perceived stress scale (PSS), a 10 item, 5-point Likert scale that evaluates the participant’s stress levels in relation to their feeling within the past month^(25)^. Validity of the scales was established in previous research^(3,26,27)^. The reliabilities were assessed by Cronbach’s alpha coefficients with very good internal consistencies of 0·859, 0·833 and 0·787 for IES-2, SCS and PSS, respectively.

The baseline survey included socio-demographic questions (age, gender, nationality, place of residence, level of education and monthly income). In addition, it included self-reported weight and height that were used to calculate the BMI. The follow-up surveys included only the anthropometric variables (weight and height) and the monthly income.

### Statistical analysis

Analysis was done based on the ‘intention to treat’ analysis using IBM SPSS version 22 (SPSS Inc.). Statistical significance was reported at the conventional level of *P* < 0·05. Participants with missing data on a scale (2·56 % of the whole sample) were excluded from the relative analysis. Normality tests were performed for continuous variables; accordingly, normally distributed data were presented as means and standard deviations and parametric testing was run. For non-parametric variables, median and inter-quartile range were reported. Categorical data were reported as counts and percentages. Descriptive statistics were performed at T0, T1 and T2. Participants’ characteristics were compared with the assigned group, using one-way ANOVA for continuous variables, and Chi-square or Fisher exact tests for categorical variables. Post-hoc comparisons were made using Bonferroni’s method. Changes in the same variables were assessed using one-way repeated measures ANOVA for continuous variables. The active and the passive IE groups were combined into one intervention group, and the analysis was repeated for intervention *v*. control groups. The effect size of the result was assessed by multivariate tests partial eta squared using the commonly used guidelines proposed by Cohen (1988, pp. 284–287): 0·01 = small, 0·06 = moderate and 0·14 = large effect size. For the categorical variable ‘Monthly income’, more than 40 % of the sample refused to answer at each assessment point. In light of the current economic crisis in Lebanon, this was a sensitive question, so no further than baseline analysis was carried for this variable. Pearson correlations were used to assess the correlation between IES-2, PSS, SCS and BMI at the different time points (T0, T1 and T2). Simple linear regressions were performed to identify the variables to be included in the multiple linear regression models. Variable with a *P*-value < 0·2 at the bivariate level was considered eligible to be included in the multiple linear regression models. Accordingly, standard multiple linear regression models were used to assess predictors of IES-2 at T0, T1 and T2. Variables were inserted manually one by one. Three models were generated. Preliminary analyses were conducted to ensure no violation of the assumptions of normality, linearity, multicollinearity and homoscedasticity assessed by the normal probability plot (P-P) of the regression-standardised residuals, the scatterplot and the tolerance and the variance inflation factor. The percentage of variations in the outcome was measured by normal R square. At T0, predictors included in the model were BMI, PSS and SCS, as well as gender. In each follow-up regression, participants’ BMI, PSS, SCS and pre-intervention IES-2 scores were entered as covariates.

## Results

### Participants characteristics

In total, 195 participants agreed to join the study. The CONSORT diagram is reported in the online supplementary material. As for the demographic characteristics, 76·4 % of the study sample were females, and 75·3 % had Bachelor’s University degree or above. The majority lived in South Lebanon (49·7 %), followed by Mount Lebanon (20 %) and Greater Beirut (12·3 %). Most of the participants joined the study in February, March and November 2021 (35·9 %, 24·6 % and 19·5 %, respectively). The median age (inter-quartile range) of the whole sample was 26 years (10) and the mean BMI was 25·46 ± 4·62 kg/m^2^. The majority of the participants did not report their monthly income (45·6 %). There were no differences between groups in any of the baseline demographic characteristics. Although not of statistical significance, active IE group participants were mostly females and were older.

### Baseline scores

The baseline PSS score for the whole sample (*n* 195) was 22·38 ± 5·80, indicating moderate stress levels^(28–30)^.The mean SCS score for the whole sample was also indicative of moderate overall SC (3·14 ± 0·42)^(31)^. As for the IES score, the whole sample had a mean of 3·25 ± 0·56. Despite being statistically insignificant, analysis by group showed that the passive IE group had lower IES total at baseline compared with both active IE and control groups (3·21 ± 0·60 *v*. 3·26 ± 0·57 *v*. 3·28 ± 0·53 respectively, *P* > 0·05). Interestingly, active IE group had more PSS scores compared with passive IE and control groups (23·40 ± 6·38 *v*. 22·26 ± 5·71 *v*. 21·49 ± 5·18, respectively, *P* > 0·05). On the other hand, control group participants had higher SCS scores compared with active and passive IE groups (3·19 ± 0·43 *v*. 3·06 ± 0·39 *v*. 3·18 ± 0·42, respectively, *P* > 0·05). Interestingly, the over-identification subscale was significantly different between groups (3·07 ± 0·83 in the control group *v*. 2·90 ± 0·67 in the passive IE group *v*. 2·62 ± 0·73 in the active IE group, *P* = 0·003) (Table 1).

**Table 1:** Baseline survey scores (T0)

Characteristics	All (*n* 195)		Active IE group (*n* 65)		Passive IE group (*n* 65)		Control group (*n* 65)		*P*
	Mean	sd	Mean	sd	Mean	sd	Mean	sd	
BMI	25·46	4·62	25·62	4·99	24·90	4·16	25·86	4·69	0·477
IES-2 scale	3·25	0·56	3·26	0·57	3·21	0·60	3·28	0·53	0·76
IES-2 subscales									
Unconditional permission to eat	3·24	0·64	3·19	0·54	3·29	0·72	3·25	0·64	0·666
Eating for physical rather than emotional reasons	3·09	0·88	3·07	0·98	2·98	0·82	3·24	0·83	0·228
Reliance on hunger and satiety cues	3·33	0·89	3·41	0·87	3·26	0·96	3·33	0·85	0·63
Body-food choice congruence	3·36	0·89	3·51	0·76	3·38	0·96	3·19	0·92	0·122
PSS	22·38	5·80	23·40	6·38	22·26	5·71	21·49	5·18	0·169
SCS	3·14 (*n* 193)	0·42	3·06	0·39	3·18 (*n* 64)	0·42	3·19 (*n* 64)	0·43	0·13
SCS subscales									
Self-kindness	3·31 (*n* 193)	0·77	3·22	0·74	3·38 (*n* 64)	0·83	3·33 (*n* 64)	0·72	0·486
Self-judgement	2·98 (*n* 193)	0·70	2·84	0·69	3·06 (*n* 64)	0·70	3·04 (*n* 64)	0·71	0·147
Common humanity	3·43 (*n* 194)	0·72	3·45	0·76	3·46 (*n* 64)	0·65	3·36	0·73	0·626
Isolation	2·98 (*n* 193)	0·76	2·88	0·73	3·00 (*n* 64)	0·73	3·05 (*n* 64)	0·82	0·408
Mindfulness	3·30 (*n* 193)	0·63	3·32	0·66	3·27 (*n* 64)	0·57	3·31 (*n* 64)	0·67	0·891
Over-identification	2·86 (*n* 193)	0·77	2·62	0·73	2·90 (*n* 64)	0·67	3·07 (*n* 64)	0·83	0·003*

IE, intuitive eating; IES-2, Intuitive Eating Scale 2.

* Stands for *P*-value < 0·05.

### Within group analysis

As for the full sample assessment, a significant improvement in IES-2 between T0 and T2 was evident (mean difference T2-T0 = +0·096, se = 0·04, *P* = 0·049). This effect was driven by a significant improvement in subscale two, eating for physical rather than emotional reasons (mean difference T2-T0 = +0·22, se = 0·057, *P* = 0·001). SCS also showed a significant improvement (mean difference T2-T0 = +0·069, se = 0·03, *P* = 0·05). In addition, PSS presented a significant reduction in the full sample between the different assessment points (mean difference T2-T0 = –1·43, se = 0·472, *P* = 0·006). As for subgroup analysis, for IES-2 scale, there was a significant effect of time, Wilks’ lambda = 0·90, F (2, 55) = 3·07, *P* = 0·05, in the passive IE group only. The effect size of the intervention explained by multivariate partial eta square was 0·101, which is considered a moderate to high effect size according to the commonly used guidelines proposed by Cohen, pp. 284 – 7^(32)^. This significant effect of time was driven by an improvement in subscale 2, eating for physical rather than hunger cues subscale, where Wilks’ lambda was 0·796, F (2, 55) = 7·06, *P* = 0·002, and the effect size was large (multivariate partial eta square = 0·204). For the SCS, there was a significant effect of time, Wilks’ lambda = 0·88, F (2, 63) = 4·40, *P* = 0·01, in the active IE group only. This indicates that SC increased significantly with time in this group. Particularly, it increased by 0·116 between T0 and T1 and was maintained by T2 (slight non-significant increase by 0·19). The effect size of the intervention was 0·123, which is considered a moderate to high effect size. For the PSS, there was a significant effect of time, Wilks’ lambda = 0·86, F (2, 63) = 5·21, *P* = 0·08, in the active IE group only. This indicates that PS decreased significantly with time in this group. In particular, it decreased by 1·4 between T0 and T1 and by 2·51 between T0 and T2. The effect size of the intervention was 0·142, which is considered of high effect size. As for BMI, the three groups showed minor non-significant reductions in BMI with time. Results were similar when the active and passive groups were considered as one intervention group; the intervention group showed a significant improvement in IES-2 scores (mean difference T2-T0 = 0·128, se = 0·052, *P* = 0·05), whereas the change in IES-2 among the control group participants was minimal (mean difference T2-T0 = 0·025, se = 0·059, *P* = 0·382). The effect size of the intervention explained by multivariate partial eta square was 0·05, which is considered a low to moderate effect size (Table 2).

**Table 2:** IES-2, IES-2 subscales, PSS, SCS and BMI scores at different time points

Outcome	Assessment point	Full sample (*n* 177)			Active IE group (*n* 65)			Passive IE group (*n* 57)			Control group (*n* 55)		
		Mean	sd	*P*	Mean	sd	*P*	Mean	sd	*P*	Mean	sd	*P*
IES-2	T0	3·27	0·55	0·049*	3·26	0·57	0·271	3·23	0·60	0·05	3·31	0·5	0·38
	T1	3·33	0·48		3·25	0·48		3·38	0·51		3·37	0·45	
	T2	3·36	0·49		3·34	0·50		3·41	0·54		3·33	0·42	
	Significant Paired Assessment	T0 *v*. T2						T0 *v*. T2					
IES-2 subscales													
Unconditional permission to eat subscale	T0	3·26	0·65	0·74	3·19	0·54	0·41	3·32	0·74	0·92	3·29	0·7	0·21
	T1	3·23	0·59		3·13	0·55		3·36	0·61		3·20	0·6	
	T2	3·24	0·62		3·23	0·58		3·35	0·72		3·16	0·57	
	Significant paired assessment												
Eating for physical rather than emotional reasons subscale	T0	3·11	0·89	0·001*	3·07	0·98	0·23	2·96	0·81	0·002*	3·30	0·82	0·18
	T1	3·23	0·81		3·10	0·84		3·16	0·77		3·44	0·78	
	T2	3·33	0·75		3·22	0·75		3·34	0·81		3·43	0·67	
	Significant paired assessment	T0 *v*. T2						T0 *v*. T2					
Reliance on hunger and satiety cues subscale	T0	3·34	0·88	0·08	3·41	0·87	0·63	3·29	0·96	0·1	3·31	0·81	0·33
	T1	3·46	0·76		3·43	0·68		3·25	0·89		3·43	0·7	
	T2	3·46	0·85		3·52	0·80		3·51	0·99		3·34	0·75	
	Significant paired assessment												
Body-food choice congruence subscale	T0	3·40	0·85	0·87	3·51	0·76	0·725	3·50	0·90	0·58	3·18	0·87	0·41
	T1	3·44	0·72		3·43	0·59		3·58	0·85		3·29	0·71	
	T2	3·42	0·81		3·46	0·78		3·48	0·93		3·32	0·71	
	Significant paired assessment												
SCS	T0	3·15	0·42	0·05	3·06	0·39	0·01*	3·19	0·4	0·32	3·21	0·45	0·88
	T1	3·20	0·40		3·17	0·42		3·23	0·34		3·20	0·45	
	T2	3·22	0·41		3·15	0·36		3·28	0·40		3·23	0·47	
	Significant paired assessment	T0 *v*. T2			T0 *v*. T1								
PSS	T0	22·43	5·76	0·006	23·40	6·38	0·008*	22·46	5·50	0·43	21·25	5·10	0·31
	T1	21·34	6·06		22·00	5·86		21·75	6·50		20·13	5·70	
	T2	21·00	5·40		20·89	5·62		21·26	6·10		20·85	4·40	
	Significant paired assessment	T0 *v*. T1, T0 *v*. T2			T0 *v*. T1, T0 *v*. T2								
BMI	T0	25·49	4·65	0·11	25·62	4·99	0·696	24·90	4·36	0·06	25·95	4·50	0·35
	T1	25·39	4·85		25·54	4·98		24·71	4·40		25·91	5·10	
	T2	25·28	4·62		25·51	4·78		24·57	4·20		25·74	4·90	
	Significant paired assessment												

Assessment points: T0: at baseline, T1: post-intervention, T2: 5 weeks follow-up. IES-2, Intuitive Eating Scale 2; PSS, perceived stress scale; SCS, self-compassion scale.

* Stands for *P* = value < 0·05.

### Predictors of Intuitive Eating Scale 2 at different time points

Results from simple linear regression revealed that at T0, PSS, SCS, BMI and gender, and at T1 and T2, PSS, SCS, BMI and baseline IES-2 were eligible to be included in the model with IES-2 as an outcome. Three multiple linear regressions models were performed (Table 3). The model generated at T0 explained 24·7 %, at T1 explained 50 % and at T2 explained 31·7 % of the variations in IES-2. At T0, PSS, SCS, BMI and gender were found to be significant predictors of IES-2, with BMI being the best predictor (beta = –0·273, *P* < 0·0001). At T1, PSS, BMI and baseline IES-2 scores were found to be significant predictors of IES-2 at T1, with baseline IES-2 score being the best predictor (beta = 0·608, *P* < 0·0001). Finally, at T2, SCS and baseline IES-2 scores were found to be significant predictors of IES-2, with baseline IES-2 score being the best predictor (beta = 0·411, *P* < 0·0001).

**Table 3:** Multiple linear regression between independent variables and IES-2 at different time points

Assessment point	Dependent variable	Predictors	Unstandardised beta	95 % CI	Standardised beta	*P*	Model summary (*R* ^2^)
T0	IES-2	PSS	−0·021	–0·036, –0·006	−0·217	0·006*	0·247
		SCS	0·278	0·08, 0·476	0·209	0·006*	
		BMI	−0·033	–0·048, –0·018	−0·273	< 0·0001**	
		Gender	−0·258	–0·429, –0·087	−0·196	0·003*	
T1		PSS	−0·013	–0·022, –0·003	−0·155	0·012*	0·5
		SCS	0·086	–0·062, 0·234	0·069	0·255	
		BMI	−0·013	–0·024, –0·002	−0·125	0·023*	
		Baseline IES-2	0·534	0·438, 0·630	0·608	< 0·0001**	
T2		PSS	−0·004	–0·016, –0·009	−0·039	0·564	0·317
		SCS	0·337	0·174, 0·5	0·278	< 0·0001**	
		BMI	−0·005	–0·018, –0·009	−0·043	0·498	
		Baseline IES-2	0·364	0·250, 0·478	0·411	< 0·0001**	

IES-2, Intuitive Eating Scale 2; PSS, perceived stress scale; SCS, self-compassion scale.

* Stands for *P*-value less than 0·05.

** Stands for *P*-value less than 0·0001.

## Discussion

The present randomised controlled trial aimed to assess the effectiveness of a text message-based IE programme, on IE in Lebanese adults, while correcting for SC, PS and BMI. The findings suggest an improvement in IE among intervention groups.

### Major findings

Results confirmed the first hypothesis as both groups receiving IE-based text messages showed an improvement in overall IE as compared with control. However, unlike what was hypothesised, the passive IE group showed higher improvement in IE compared with the active group, driven by significant enhancement in the ‘eating for physical rather than emotional reasons’ subscale at the second follow-up. The fact that the particular subscale is involved could be due to individuals who are new to IE taking the idea of being mindful to physical cues as a ‘hunger-fullness’ diet, which could be very easy to remember after the messages were sent. The findings hence suggest that sending only the messages of IE without further active engagement may be more effective in improving IE, and the effect of the intervention needs time to be evident. In addition, the results suggest that the passive IE group showed better improvement in IE compared with the active. This could possibly be justified by the practice exercises reminding the participants of their habits instead of trying to engage them with the principle; especially that previous research used active engagement, in the form of questions and tips, to remind the participants about the learnt skills^(22)^. It could be useful for future research to use exercises that would reassure the IE principle instead of assessing previous habits. Moreover, the present study confirmed that gender predicts IE. Indeed, females had significantly lower IE scores compared with males. This could be possibly explained by the higher stigma and the internalisation of thin ideal that females are subject to, which in turn influence their dieting behaviour and body esteem^(33)^. In addition, studies suggest that females are more likely to use food to manage their emotions while males usually have higher body appreciation, which is a predictor of IE^(34–37)^. Interestingly, gender lost significance at follow-ups suggesting that the intervention attenuated gender-related differences in IE.

### Intervention effect on intuitive eating

In an IE-based text messages intervention study conducted in college students in Midwestern University, IES total scores significantly increased in the text messages group, specifically due to improvement in the reliance on hunger and satiety cues^(21)^. Similarly, in the current sample, there was a significant improvement in IES-2 post-intervention and at 5 weeks follow-up, driven by a significant increase in eating for physical rather than emotional reasons. It is worth noting that the results of this paper showed that IES-2 significantly improved in the full sample; hence, we could hypothesise that improvements in nutrition-related behaviours could be related to texting programmes that increase nutrition/health knowledge in general. For example, the Mobile Myplate intervention, which aimed to assess the effectiveness of nutrition-related messages on college students’ knowledge and behaviour *v*. email handouts, showed that this approach is helpful in promoting positive nutrition-related behaviours^(38)^. The success of such programmes validates that texting interventions could be cost-effective and easy to administer behaviour-changing interventions, especially that participants do not have to seek and search for the information, they receive it promptly. In addition to the significant increase in total IE scores, this study showed that IES-2 subscale 2, ‘eating for physical rather than emotional reasons’, also increased significantly among the full sample, and particularly the passive IE group which was the only to show a significant improvement at the second follow-up. This finding may indicate that the text messages acted as a good reminder to keep the participants conscious to the difference between physical hunger and emotional hunger. Hence, this may convey that nutrition-related messages, and in particular, IE-related messages would stimulate consciousness towards body hunger cues.

### Intervention effect on independent variables perceived stress, self-compassion and BMI

The current IE text-messaging intervention had a positive impact on PS. The findings suggest that IE-based interventions could have a positive impact on the management of PS. IE principles might have led to a better stress management, even in populations under stress like our sample, given the economic deterioration, health crisis and political combats that are currently being faced in Lebanon^(39,40)^. This finding is in line with other studies correlating IE with stress. For example, in a cross-sectional analysis of an interventional study in Finland, individuals with higher PS reported less IE^(41)^. However, intervention studies reported different findings concerning IE intervention’s effect on stress. Despite the significant correlation between IE and PS, Loughran *et al*.’s IE-based text-messaging intervention did not associate with post-intervention PSS. Moreover, the intervention group showed an increase in PS from pre to post-intervention, which opposes our findings^(21)^. Authors suggested that the observed increase in PS was associated with the environment and workload of the college students under study^(21)^. However, a possible justification for this result could be the fact that the IE messages they delivered did not include coping strategies and practical tips. In this context, a randomised controlled trial used a non-dieting stress reduction programme, offering mind–body relaxation techniques (mindfulness, meditation etc.) to raise awareness of thoughts and emotions and to support the development of healthy coping strategies^(42)^. This programme led to significant increase in IE and great improvement in stress management behaviours suggesting that interventions addressing stress and mindfulness are effective in eliciting behaviour change^(42)^. In line with this, our findings suggest that participants used the received IE-related content as a coping mechanism against stress.

This particular intervention is noteworthy given that it significantly improved SC in the intervention groups. Along with this positive improvement, a positive correlation was observed between SC and IE at all assessment points, and at the last follow-up, SC became the strongest predictor of IE. Research in this field documented the effect of SC on IE by highlighting how being more self-compassionate towards internal body experiences may enhance the ability to eat intuitively^(43)^. However, no previous work studied the effect of IE interventions on SC. Interventional studies on SC demonstrated that the latter is a skill that can be learned to influence eating behaviours and other health outcomes^(44)^. Hence, SC interventions included mindfulness and mindful eating, meditation and yoga, developing a compassionate inner voice, adopting SC in daily life and managing emotions among others^(44)^. The novel addition of our study is that IE-based text-messaging interventions can be used as a method to enhance SC. The positive effect observed could be because IE principles focus a lot on connecting to body cues, attending to the soft inner voice and managing emotions without using food. Thus, IE principles acted as a means to relate the individual to his or her inner self and to stimulate his or her feelings of body appreciation, security and safety, which ideally should enhance SC^(45,46)^. This is also confirmed by studies suggesting that SC influences brain centres related to threat, feeling and behaviours^(45–48)^. Such messages are in line with the SC interventions provided by the literature.

It was previously demonstrated in observational studies that IE is negatively correlated with BMI^(13)^ and in interventional studies that IE interventions could maintain BMI^(19,20)^. The current study documented this negative correlation between IE and BMI. However, prior to the intervention, BMI was the strongest predictor of IE. This had changed in the follow-ups and BMI lost significance as a predictor. In other words, it can be inferred that the effect the intervention had on IE attenuated the effect of BMI on IE. Further research is needed to elucidate the actual effect. Despite that, combining IE principles to reduce external eating, along with educating about what to eat in response to internal cues in terms of healthy food choices, can provide optimal health outcomes. This should be considered in future research given that diet quality was not improved following IE intervention in previous research^(18)^.

### Strengths and limitations

The current study has several strengths and limitations. Strengths include its randomised interventional design, which reduces allocation and sampling biases and allows comparison to determine the actual effect. Furthermore, this study was blinded to the participants, hence maximising the validity of the results. In addition, the study is novel in the region and in the way it looked at different interacting variables, and their influence on eating behaviours. Moreover, the design allowed to deliver an intervention that mimics daily life exposure to nutrition awareness through social media without manipulation of the participants’ setting and environment.

Limitations of the study include its small sample size when compared with other text-messaging interventions and the use of self-reported surveys. Furthermore, the method of recruitment (snowball method) is a significant limitation that could have created a non-representable sample. Other limitations include the level of education and place of residence of the participants, with the majority being educated, and residing in South Lebanon, as data were collected using a snowball method. The possibility of cross contamination, where participants would share messages with each other, is also a significant limitation; however, we could not guarantee that the participants know or contact each other. In addition, residual confounders that are known to influence IE such as chronic diseases, mental health, eating habits, self-efficacy, social gathering and physical activity level were not measured in an effort not to over-burden participants.

## Conclusion

The present study showed that IE-based text messages may help in enhancing IE, reducing PS and improving SC. The results may serve as a stepping stone for future IE interventions targeting larger sample to enhance all the three outcomes, which can, thanks to the ease of text messaging, be widespread on a public health level to inform awareness campaigns. Future studies may benefit from investigating the above-studied variables in different age groups and in clinical populations (e.g. eating disorders, anxiety disorders, etc.). Despite that, the empirical findings of this study are remarkable in that they represent the first study to implement the concept of active and passive engagement in texting programmes, and to explore the effect of IE intervention on SC and PS, which might be of clinical significance in health programmes.

## Supplementary material

For supplementary material accompanying this paper visit https://doi.org/10.1017/S1368980023000939. click here to view supplementary material

## References

[1] Tribole E & Resch E (2012) Intuitive Eating. New York city, USA: Macmillan.

[2] Tribole E & Resch E (2003) Intuitive Eating: A Revolutionary Program That Works. New York: St. Martin’s Griffin.

[3] Tylka TL & Kroon Van Diest AM (2013) The Intuitive Eating Scale–2: item refinement and psychometric evaluation with college women and men. J Counsel Psychol 60, 137. 10.1037/a0030893 23356469

[4] Barraclough EL , Hay-Smith EJC , Boucher SE et al. (2019) Learning to eat intuitively: a qualitative exploration of the experience of mid-age women. Health Psychol Open 6, 2055102918824064. 30746153 10.1177/2055102918824064 PMC6360478

[5] Pannicke B , Kaiser T , Reichenberger J et al. (2020) Networks of stress, affect and eating behaviour: anticipated stress coping predicts goal-congruent eating. Int J Behav Nutr Phys Act 18, 1–14. 10.1186/s12966-020-01066-8 PMC7796605 33422046

[6] Madden CE , Leong SL , Gray A et al. (2012) Eating in response to hunger and satiety signals is related to BMI in a nationwide sample of 1601 mid-age New Zealand women. Public Health Nutr 15, 2272–2279. 22443858 10.1017/S1368980012000882 PMC10271589

[7] Bruce LJ & Ricciardelli LA (2016) A systematic review of the psychosocial correlates of intuitive eating among adult women. Appetite 96, 454–472. 26474781 10.1016/j.appet.2015.10.012

[8] Dalen J , Smith BW , Shelley BM et al. (2010) Pilot study: mindful Eating and Living (MEAL): weight, eating behavior, and psychological outcomes associated with a mindfulness-based intervention for people with obesity. Compl Ther Med 18, 260–264. 10.1016/j.ctim.2010.09.008 21130363

[9] Tylka TL (2006) Development and psychometric evaluation of a measure of intuitive eating. J Couns Psychol 53, 226. 10.1037/a0030893 23356469

[10] Cardoso A , Oliveira S & Ferreira C (2020) Negative and positive affect and disordered eating: the adaptive role of intuitive eating and body image flexibility. Clin Psychol 24, 176–185.

[11] Burnette CB (2020) An Intuitive Eating Intervention for College Women With Disordered Eating: Evaluating Two Accessible and Affordable Approaches. https://scholarscompass.vcu.edu/cgi/viewcontent.cgi?article=7220&context=etd (accessed April 2023).

[12] Smith T & Hawks SR (2006) Intuitive eating, diet composition, and the meaning of food in healthy weight promotion. Am J Health Education 37, 130–136.

[13] Van Dyke N & Drinkwater EJ (2014) Review article relationships between intuitive eating and health indicators: literature review. Public Health Nutr 17, 1757–1766. 23962472 10.1017/S1368980013002139 PMC10282369

[14] Allen AB & Leary MR (2010) Self-compassion, stress, and coping. Soc Pers Psychol Compass 4, 107–118. 10.1111/j.1751-9004.2009.00246.x PMC2914331 20686629

[15] Finlay-Jones AL , Rees CS & Kane RT (2015) Self-compassion, emotion regulation and stress among Australian psychologists: testing an emotion regulation model of self-compassion using structural equation modeling. PLoS One 10, e0133481. 26207900 10.1371/journal.pone.0133481 PMC4514830

[16] Neff KD & Germer CK (2013) A pilot study and randomized controlled trial of the mindful self-compassion program. J Clin Psychol 69, 28–44. 23070875 10.1002/jclp.21923

[17] Jayne JM , Ayala R , Karl JP et al. (2020) Body weight status, perceived stress, and emotional eating among US Army Soldiers: a mediator model. Eating Behav 36, 101367. 10.1016/j.eatbeh.2020.101367 32018191

[18] Grider HS , Douglas SM & Raynor HA (2021) The influence of mindful eating and/or intuitive eating approaches on dietary intake: a systematic review. J Acad Nutr Dietetics 121, 709–727. 10.1016/j.jand.2020.10.019 33279464

[19] Bacon L , Stern JS , Van Loan MD et al. (2005) Size acceptance and intuitive eating improve health for obese, female chronic dieters. J Am Dietetic Assoc 105, 929–936. 10.1016/j.jada.2005.03.011 15942543

[20] Boucher S , Edwards O , Gray A et al. (2016) Teaching intuitive eating and acceptance and commitment therapy skills via a web-based intervention: a pilot single-arm intervention study. JMIR Res Protocol 5, e5861. 10.2196/resprot.5861 PMC5086025 27742602

[21] Loughran TJ , Harpel T , Vollmer R et al. (2018) Effectiveness of intuitive eating intervention through text messaging among college students. Coll Stud J 52, 232–244.

[22] Bauer S , de Niet J , Timman R et al. (2010) Enhancement of care through self-monitoring and tailored feedback via text messaging and their use in the treatment of childhood overweight. Patient Educ Counsel 79, 315–319. 10.1016/j.pec.2010.03.014 20418046

[23] Tribole E & Resch E (2017) The Intuitive Eating Workbook: Ten Principles for Nourishing a Healthy Relationship with Food. Oakland, CA: New Harbinger Publications.

[24] Neff KD (2003) The development and validation of a scale to measure self-compassion. Self Identity 2, 223–250.

[25] Cohen S , Kamarck T & Mermelstein R (1994) Perceived stress scale. Measuring Stress: Guide Health Soc Sci 10, 1–2.

[26] Alabdulaziz H , Alquwez N , Almazan JU et al. (2020) The Self-Compassion Scale Arabic version for baccalaureate nursing students: a validation study. Nurse Educ Today 89, 104420. 32276172 10.1016/j.nedt.2020.104420

[27] Chaaya M , Osman H , Naassan G et al. (2010) Validation of the Arabic version of the Cohen Perceived Stress Scale (PSS-10) among pregnant and postpartum women. BMC Psychiatr 10, 111. 10.1186/1471-244X-10-111 PMC3016315 21159169

[28] Bhat RM , Sameer M & Ganaraja B (2011) Eustress in education: analysis of the perceived stress score (PSS) and blood pressure (BP) during examinations in medical students. J Clin Diagn Res 5, 331–1335.

[29] Thangaraj S & D’souza L (2014) Prevalence of stress levels among first year medical undergraduate students. Int J Interdisciplinary Multidisciplinary Stud (IJIMS) 1, 176–181.

[30] Swaminathan A , Viswanathan S , Gnanadurai T et al. (2016) Perceived stress and sources of stress among first-year medical undergraduate students in a private medical college–Tamil Nadu. Natl J Physiol Pharm Pharmacol 6, 9–14.

[31] Neff K (2003) Self-compassion: an alternative conceptualization of a healthy attitude toward oneself. Self Identity 2, 85–101.

[32] Cohen J (1988) Statistical Power Analysis for the Behavioral Sciences. Hillsdale, NJ: Lawrence Erlbaum Associates. pp. 20–26.

[33] Kleppe A (2020) Exploring intuitive eating in the adolescent population. Master’s paper. doi: 10.17615/z0dj-ev40.

[34] Andrew R , Tiggemann M & Clark L (2015) Predictors of intuitive eating in adolescent girls. J Adolesc Health 56, 209–214. 25620304 10.1016/j.jadohealth.2014.09.005

[35] Andrew R , Tiggemann M & Clark L (2016) Predictors and health-related outcomes of positive body image in adolescent girls: a prospective study. Dev Psychol 52, 463. 26727595 10.1037/dev0000095

[36] Lemoine J , Konradsen H , Jensen AL et al. (2018) Factor structure and psychometric properties of the Body Appreciation Scale-2 among adolescents and young adults in Danish, Portuguese, and Swedish. Body Image 26, 1–9. 29772464 10.1016/j.bodyim.2018.04.004

[37] Luo Y-J , Niu G-F , Kong F-C et al. (2019) Online interpersonal sexual objectification experiences and Chinese adolescent girls’ intuitive eating: the role of broad conceptualization of beauty and body appreciation. Eating Behav 33, 55–60. 10.1016/j.eatbeh.2019.03.004 30927695

[38] Brown ON , O’Connor LE & Savaiano D (2014) Mobile MyPlate: a pilot study using text messaging to provide nutrition education and promote better dietary choices in college students. J Am Coll Health 62, 320–327. 24654921 10.1080/07448481.2014.899233

[39] Bizri M , Kassir G , Tamim H et al. (2021) Psychological distress experienced by physicians and nurses at a tertiary care center in Lebanon during the COVID-19 outbreak. J Health Psychol 27, 1288–1300. 33567926 10.1177/1359105321991630 PMC7879044

[40] Mourani SC & Ghreichi M-C (2021) Mental Health Reforms in Lebanon During the Multifaceted Crisis. https://www.arab-reform.net/publication/mental-health-reforms-in-lebanon-during-the-multifaceted-crisis/ (accessed April 2023).

[41] Järvelä-Reijonen E , Karhunen L , Sairanen E et al. (2016) High perceived stress is associated with unfavorable eating behavior in overweight and obese Finns of working age. Appetite 103, 249–258. 27108837 10.1016/j.appet.2016.04.023

[42] Katzer L , Bradshaw AJ , Horwath CC et al. (2008) Evaluation of a ‘nondieting’ stress reduction program for overweight women: a randomized trial. Am J Health Promot 22, 264–274. 18421891 10.4278/060728113R1.1

[43] Schoenefeld SJ & Webb JB (2013) Self-compassion and intuitive eating in college women: examining the contributions of distress tolerance and body image acceptance and action. Eating Behav 14, 493–496. 10.1016/j.eatbeh.2013.09.001 24183143

[44] Ferrari M , Hunt C , Harrysunker A et al. (2019) Self-compassion interventions and psychosocial outcomes: a meta-analysis of RCTs. Mindfulness 10, 1455–1473.

[45] Gilbert P (2005) Compassion: Conceptualisations, Research and Use in Psychotherapy. Hove, East Sussex: Routledge.

[46] Kelly AC & Stephen E (2016) A daily diary study of self-compassion, body image, and eating behavior in female college students. Body Image 17, 152–160. 27081748 10.1016/j.bodyim.2016.03.006

[47] Depue RA & Morrone-Strupinsky JV (2005) A neurobehavioral model of affiliative bonding: implications for conceptualizing a human trait of affiliation. Behav Brain Sci 28, 313–349. 16209725 10.1017/S0140525X05000063

[48] LeDoux J (1998) The Emotional Brain: The Mysterious Underpinnings of Emotional Life. New York: Simon and Schuster.

