# Supplementary material for: Effectiveness of a text-messaging intervention on intuitive eating: a randomised controlled trial
Source: Public Health Nutr. 2023 May 11;26(8):1576–84. doi: 10.1017/S1368980023000939 (PMC10410380; doi:10.1017/S1368980023000939)
Supplement: Supplementary file 1 [file S1368980023000939sup.zip › S1368980023000939sup001.docx]

**Effectiveness of Text Messaging Intervention on Intuitive Eating: A Randomized Control trial**

# **Detailed Methodology**

This randomized control trial (RCT) recruited the participants and delivered the intervention fully online. The primary end-point of the study is IE and was measured by the intuitive eating scale (IES-2) (1). Secondary end-points included SC, measured by self-compassion scale (SCS) (2), and PS measured by perceived stress scale (PSS) (3). The questionnaires were filled via Google Form and the intervention content for all the groups was communicated via Whatsapp®.

## Study Design and Participants Recruitment

This study was a single blinded RCT with a parallel design approved by the Lebanese American University (LAU) Institutional Review Board (IRB) on February 10, 2021 and registered in the Lebanese Clinical Trials registry (LBCTR2022045031). Data collection took place between February 2021 and January 2022. Potential participants were identified and recruited using the snowball effect method; sharing the advertisement material on different social media platforms mainly Facebook®, Instagram® and Whatsapp®. Additionally, fellow dietitians and colleagues were contacted to share the call for freewill volunteers to join the study. The study randomized the participants into three groups.

## Inclusion/Exclusion Criteria

Inclusion criteria included: (1) men and women, (2) living in Lebanon, (3) aged between 18 and 50 years inclusive, (4) must have a smart phone with an active WhatsApp® account. The age group was chosen based on previous research where this group is more prone to engage in weight-loss behaviors. Participants were excluded if they were less than 18 years or more than 50 years, currently enrolled in a weight loss program, pregnant or taking medications known to affect body weight/appetite. Phone numbers of the participants were obtained upon online consent.

## Sample Size Calculation

Sample size was calculated based on the effect size of web-based IE intervention study (4). The mean intuitive eating scale change in previous research was 0.94 ± 0.67, which is indicative of a medium to high effect size as measured by Cohen`s d (5). At a power of 80% and probability of 0.05 at two-sided hypothesis different from zero, we needed 65 participant per arm. Since we have three arms, we will need a sample size of 195 participant. This sample size is suitable for the analysis plan we want to consider.

## Randomization

Randomization took place between February 2021 and November 2021. Upon consent, participants were randomized to an Active Intervention Group (AI), Passive Intervention Group (PI) and a Control Group (CG).In view of the sample size calculation, to avoid imbalances, and to produce a better estimate of treatment effect, randomization was based on permuted block sampling with six participants per block with an allocation ration of 1:1:1. Sequence generation was performed via online software: Web site: Randomization.com. 2020. ([www.randomization.com](http://www.randomization.com)). The participants were blinded for allocation and study objectives until the end of the study period.

## Procedures

The study coordinator launched the call for study registration and was responsible for the data collection and execution of the intervention. Participants willing to get involved were asked to sign an online informed consent via Google Forms, where they were to add their phone numbers and provide electronic consent. Once consented, participants were able to fill in the baseline survey. Every consented participant was then randomized into one of the three groups as mentioned earlier.

Consented participants then received ten messages over five weeks, at a rate of two messages per week, i.e. every Monday and Thursday starting the first week after consent. An additional online survey (Follow-up 1) was administered right at the end of the intervention (i.e. after week five) and then again five weeks post-intervention (follow-up 2). Then, after the second follow-up, the participant, irrespective of allocated group, received a debriefing video that explains IE principles with practical tips and examples.

Each survey required 15 minutes to be completed. All the surveys, along with the text messages content were available in both English and Arabic. At the end of the data collection period in January 2022, an online focus group was formed to get their open-ended feedback on how the intervention felt, and how it would be made better as this is the first of its kind in Lebanon. The focus group was through Zoom application and was video recorded. All study participants who mentioned their interest to join the video-recorded focus group in the consent form were contacted, 15 agreed to join and were invited to the Zoom meeting. The details of the study timeline are presented in figure 1.

The three groups were as follows:

1. **The control group** received ten general health related messages.
2. **The passive IE group** received ten messages that contain IE principles.
3. **The active IE group** received the ten principles over five weeks, additionally, every Tuesday and Thursday (one day after message receipt); they were asked to answer one multiple-choice question/practice exercise related to the principle sent earlier, to assess active involvement in the intervention. Practice exercises were adapted from the IE workbook (6).

Study participants were informed of their rights to withdraw from the study at any time point if they were overwhelmed by the rate of text messages delivery. This can happen by texting STOP to the number that they were receiving the text messages through. Based on the study timeline, text messages were sent during weekdays to spare the weekends for the participants and to avoid any pressure or load.

The study did not involve physical interaction with the participants, and did not give any new diet plan nor pills. Hence, risks are not more than minimal. Discomfort might happen though, especially from the text messages. This was avoided by sending only two messages per week, which were separated, and sent only on weekdays to spare weekends for the participants. The benefits of the study include increase in knowledge and awareness. Especially after the debriefing, participants were able to learn a new helpful technique that would help them appreciate their body and create a healthy relationship with food.

## Measurement Instruments

Unless indicated otherwise, participants` assessment via the online survey was evaluated at all points for the three groups; at baseline (T0), at the end of the intervention for follow-up 1 (T1) and after five weeks for follow-up 2 (T2) (Figure 1).

Below is a description of the questionnaires used in the survey.

The primary outcome of the study is IE. It was assessed through the Intuitive Eating Scale 2 (IES-2) embedded in the surveys (1). It is a 23-item self-reported scale used to assess the eating habits of individuals over the past month. It is composed of four sub-scales: the unconditional permission to eat, eating for physical rather than emotional reasons, dependence on hunger and satiety cues, and the congruence between body and food. The scale uses a five point Likert scale, with one being strongly disagree and five being strongly agree. The score is computed by adding the items of the scale, after reversing negatively scored items, and averaging the computed value through dividing it by the total number of items. Hence, the IES-2 total score ranges between zero and five, and thus getting higher scores means higher IE practices (1). An example of an item in the questionnaire: item one, “I try to avoid certain foods high in fat, carbohydrates, or calories”.

Secondary outcomes are PS and SC, and their scales were also embedded in the survey. SC was assessed using self-compassion scale (SCS) (2). It is composed of 26 items that evaluate SC under six subscales, over-identification items, self-judgment items, self-kindness items, common humanity items, isolation items and mindfulness items. It uses a five point Likert scale, with one being almost never and five being almost always (2), and evaluates how often the participant would behave in the stated manner. For example, item 2 in the SCS is “When I am feeling down, I tend to obsess and fixate on everything that is wrong”. The total score is calculated by adding the score of each item, after reversing negatively scored items, and dividing the number obtained by the number of items. Hence, the SCS total score ranges between zero and five, and thus getting higher scores will mean better SC. Usually, scores 1 – 2.49 are considered low, between 2.5 – 3.5 are considered moderate and 3.51 – 5.0 are considered high (2).

PS was measured by Perceived Stress Scale (PSS) (3). PSS is a 10 item scale that evaluates the participant`s stress levels in relation to their feeling within the past month. It uses a five-point Likert scale (0: never; 4: very often). To obtain a total score, positively scored items were reversed, and then the items are summed to obtain a total PSS score, with values ranging from “0” to “40”. The higher the score, the higher the PS level. For example, item 3 of the PSS is “In the last month, how often have felt nervous and stressed?” Scores ranging 0 – 13 are considered low stress, 14 – 26 are considered moderate stress and 27 – 40 are considered high stress (7-9).

Other demographic data were collected. The baseline survey included demographic questions (age, gender, nationality, place of residence, level of education and monthly income). In addition, it included self-reported weight and height that were used to calculate the BMI. The follow-up surveys included only the anthropometric variables (weight and height) and the monthly income along with the scales.

## Validity and Reliability of Questionnaires

The IES – 2 is valid (1) for use among adults. In addition, the scale was translated into Arabic, and back translated into English. It was presented to two Professors to read and validate. The scale was then pilot tested over 20 volunteers who have similar characteristics of the study participants to assess its readability. As for reliability, according to Camilleri et al (2015), the IES-2 scale showed satisfactory reliability; with a Cronbach alpha coefficient reported of 0.85 (10). In the current study, the Cronbach alpha coefficient was 0.859, which represents a very good internal consistency reliability for the scale. As for subscales, Cronbach alpha coefficient was 0.61, 0.86, 0.90, and 0.84 for the unconditional permission to eat subscale, eating for physical rather than emotional reasons subscale, reliance on hunger and satiety cues subscale and body-food choice congruence subscale respectively.

The validated and reliable Arabic version of the SCS that adopted the translation/back translation method procedures was used as it previously showed appropriate psychometric properties (11, 12). As for reliability, the SCS Arabic version showed good reliability; with a Cronbach alpha coefficient of 0.86 for the whole scale, and the values for the six subscales were from 0.76 to 0.85 (12). In the current study, the Cronbach alpha coefficient for the scale was 0.833 indicating a satisfactory internal consistency reliability for the scale. As for subscales, Cronbach alpha coefficient was 0.79, 0.70, 0.73, 0.64, 0.73, and 0.7 for the self-kindness items, self-judgment items, common-humanity items, isolation items, mindfulness items, and over-identification items respectively.

The valid Arabic version of PSS-10 was used as it previously demonstrated adequate psychometric properties (13). According to the authors, the internal consistency reliability of the Arabic PSS-10 was 0.74. In the current sample, the Cronbach alpha coefficient was 0.787 indicating a good internal consistency reliability for the scale. For this scale, the mean inter-item correlation value was 0.267, which is optimal.

## Ethical Considerations

Ensuring a safe environment and respecting ethical beliefs are both priority. The study obtained approval from the Institutional Review Board at LAU. The study details were well explained to the participants through the consent form which was submitted online via Google forms, where potential participants had the chance to read and provide online signature and phone numbers expressing willingness to join. They were provided with the phone number of the study coordinator and the email address of the PI if they require further clarifications. Finally, participants were informed that they have the free will to choose participation, also, that they can withdraw from the study at any point.

The control group was receiving the general health tips, which does not involve IE or eating habits modifications.

The only concern is blinding, where the main objective of the study was not communicated with the participants to prevent possible bias. Accordingly, after study completion, all participants irrespective of allocation received the debriefing video discussing the study objectives and the IE principles.

## Data Management and Efficacy Assessment

All data was collected online. Hence, data was stored in a locked computer, where only the study coordinator who is collecting the data had access to the computer. Participants’ information and contact details will be kept available for three years after study completion. After that, it will be deleted from the computer. Participants were given a research number, accordingly, they were de-identified. The participants provided their names in the consent forms and data collection forms so that the surveys will be linked to them, however, they were provided with a research number that includes their initials, group (C: control, A: active intervention and P: passive intervention), and their research number. In another word, when a participant joined the study, the study coordinator created the code, and the SPSS data entry file had this code. This was made to make sure that the research coordinator only has access to the names of participants. De-identified data will be kept for 15 years after study completion. In the focus group, participants who provided a consent to join were asked to add nicknames to their Zoom accounts if they wish too, so that names were masked, and they had the freewill to choose if they want to turn on the cameras.

The research team did not meet with the participants, and their contact information were not communicated with other agencies. Information hence was and will be kept confidential.

Efficacy of the study was assessed at the end of the intervention over two levels. First, at the level of participants where a focus group was created as mentioned earlier to obtain their feedback. Second, an objective efficacy assessment was gained from the analysis of the results.

**Focus Group:**

English Script/Questions:

We are meeting to discuss the intervention study that you had completed. The study aim was to assess the effectiveness of a five-week text-message-based IE intervention on IE, while correcting for self-compassion and perceived stress level.

1- How did you feel about the intervention?

2- What things could you modify in this intervention to make it better?

3- Was the frequency of messages suitable?

4- How did you find the length of the messages?

5- How interesting were they?

6- What do you think of the effectiveness of this type of intervention?

7- Do you have any other comments?

Thank you for joining and completing this study.

**References**

1. Tylka TL, Kroon Van Diest AM. The Intuitive Eating Scale–2: Item refinement and psychometric evaluation with college women and men. Journal of counseling psychology. 2013;60(1):137.

2. Neff KD. The development and validation of a scale to measure self-compassion. Self and identity. 2003;2(3):223-50.

3. Cohen S, Kamarck T, Mermelstein R. Perceived stress scale. Measuring stress: A guide for health and social scientists. 1994;10:1-2.

4. Boucher S, Edwards O, Gray A, Nada-Raja S, Lillis J, Tylka TL, et al. Teaching intuitive eating and acceptance and commitment therapy skills via a web-based intervention: a pilot single-arm intervention study. JMIR research protocols. 2016;5(4):e180.

5. Thalheimer W, Cook S. How to calculate effect sizes from published research: A simplified methodology. Work-Learning Research. 2002;1:1-9.

6. Tribole E, Resch E. The intuitive eating workbook: Ten principles for nourishing a healthy relationship with food: New Harbinger Publications; 2017.

7. Bhat RM, Sameer M, Ganaraja B. Eustress in education: Analysis of the perceived stress score (PSS) and blood pressure (BP) during examinations in medical students. Journal of Clinical and Diagnostic Research. 2011;5(7):331-1335.

8. Thangaraj S, D’souza L. Prevalence of Stress Levels Among First Year Medical Undergraduate Students. 2014.

9. Swaminathan A, Viswanathan S, Gnanadurai T, Ayyavoo S, Manickam T. Perceived stress and sources of stress among first-year medical undergraduate students in a private medical college–Tamil Nadu. National Journal of Physiology, Pharmacy and Pharmacology. 2016;6(1):9-14.

10. Camilleri GM, Méjean C, Bellisle F, Andreeva VA, Sautron V, Hercberg S, et al. Cross-cultural validity of the Intuitive Eating Scale-2. Psychometric evaluation in a sample of the general French population. Appetite. 2015;84:34-42.

11. Teleb AA, Al Awamleh AA. The relationship between self compassion and emotional intelligence for university students. Current Research in Psychology. 2013;4(2):20.

12. Alabdulaziz H, Alquwez N, Almazan JU, Albougami A, Alshammari F, Cruz JP. The Self-Compassion Scale Arabic version for baccalaureate nursing students: A validation study. Nurse Education Today. 2020:104420.

13. Chaaya M, Osman H, Naassan G, Mahfoud Z. Validation of the Arabic version of the Cohen Perceived Stress Scale (PSS-10) among pregnant and postpartum women. BMC psychiatry. 2010;10(1):111.
