# Supplementary material for: Effectiveness of a text-messaging intervention on intuitive eating: a randomised controlled trial
Source: Public Health Nutr. 2023 May 11;26(8):1576–84. doi: 10.1017/S1368980023000939 (PMC10410380; doi:10.1017/S1368980023000939)
Supplement: Supplementary file 1 [file S1368980023000939sup.zip › S1368980023000939sup002.docx]

Figure 1: Research Timeline

T0: Baseline, T1: Follow-up 1 at the end of the intervention, T2: Follow-up 2 after five weeks.


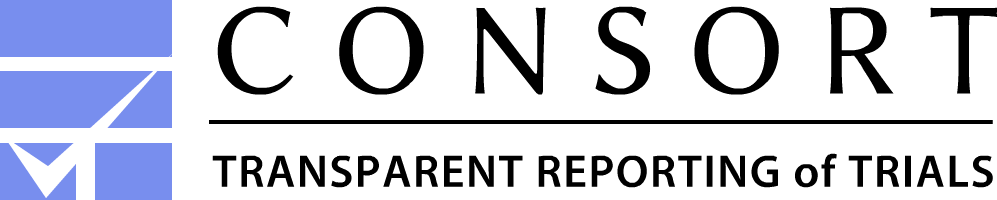


**Enrollment**

Allocated to Control (n=65)

 Received allocated intervention (n=65)

Figure 2*: CONSORT 2010 Flow Diagram*

Analysed (n= 55)

Analysed (n= 65)

Analysed (n= 57)

**Analysis**

Lost to follow-up (n=7)

 Did not respond to filling the survey

**Follow-Up T2**

Lost to follow-up (n=0)

Lost to follow-up (n=7)

 Did not respond to filling the survey

Lost to follow-up (n=0)

Lost to follow-up (n=4)

 Did not respond to filling the survey

Lost to follow-up (n=4)

 Did not respond to filling the survey

**Follow-Up T1**

Allocated to Passive IE Intervention (n=65)

 Received allocated intervention (n=65 )

Excluded (n=7)

 Not meeting inclusion criteria (n= 1)

 Declined to participate (n= 6)

Assessed for eligibility (n= 202)

Randomized (N=195)

Allocated to Active IE Intervention (n=65)

 Received allocated intervention (n=65)

**Allocation**
